# Early resumption of sexual activity following voluntary medical male circumcision in Botswana: A qualitative study

**DOI:** 10.1371/journal.pone.0186831

**Published:** 2017-11-14

**Authors:** Jenny H. Ledikwe, Nankie M. Ramabu, Lisa P. Spees, Scott Barnhart, Conrad Ntsuape, Bazghina-werq Semo, Kathleen E. Wirth

**Affiliations:** 1 Department of Global Health, University of Washington, Seattle, Washington, United States of America; 2 Botswana International Training and Education Center for Health (I-TECH), Gaborone, Botswana; 3 The Cecil G. Sheps Center for Health Services Research, University of North Carolina at Chapel Hill, Chapel Hill, NC, United States of America; 4 Department of HIV/AIDS Prevention and Care, Botswana Ministry of Health, Gaborone, Botswana; 5 Department of Epidemiology, Harvard T.H. Chan School of Public Health, Boston, Massachusetts, United States of America; 6 Department of Immunology and Infectious Diseases, Harvard T.H. Chan School of Public Health, Boston, Massachusetts, United States of America; Katholieke Universiteit Leuven Rega Institute for Medical Research, BELGIUM

## Abstract

Unprotected sexual intercourse after undergoing voluntary medical male circumcision but prior to complete wound healing can lead to major adverse events including HIV acquisition. To better understand perceptions related to early resumption of sex prior to wound healing, 27 focus group discussions were conducted among 238 adult men, women, and community leaders in Botswana. Median age among all participants was 31 years of whom 60% were male and 51% were either employed and receiving salary or self-employed. Only 12% reported being currently married. Pain, not risk of HIV acquisition, was perceived as the main adverse consequence of early resumption of sex. In fact, no participant mentioned that early resumption of sex could lead to an increase in HIV risk. Demonstrating masculinity and virility, fear of losing female partners, and misperception about post-operative wound healing also played key roles in the decision to resume sex prior to complete wound healing. Findings from this study highlight a potentially widespread lack of awareness of the increased risk of HIV acquisition during the wound healing period. Strengthening post-operative counseling and identifying strategies to discourage the early resumption of sex will be increasingly important as older men and HIV-positive men seek voluntary medical male circumcision services.

## Introduction

The World Health Organization (WHO) and the joint United Nations program on HIV/AIDS (UNAIDS) recommended voluntary medical male circumcision (VMMC) as an add-on strategy for HIV prevention in 2007 [1]. This recommendation was prompted by evidence from three randomized clinical trials demonstrating that VMMC reduces the risk of acquiring HIV infection by 51% to 60% among heterosexual men [2–4]. Since 2007, there have been substantive efforts to expand VMMC interventions in countries with high HIV prevalence and low rates of male circumcision [5, 6]. Mathematical modeling suggests that rapid scale-up of VMMC in high HIV prevalence settings is a cost-effective intervention to reduce population-level HIV transmission [7–10].

WHO/UNAIDS recommends sexual abstinence for six weeks following the VMMC procedure to ensure complete wound healing [1]. Resumption of sexual activity prior to the recommended abstinence period can delay wound healing, increase the risk of adverse events, and increase the risk of HIV acquisition [11–13]. During the three randomized clinical trials that demonstrated the efficacy of VMMC [2–4], 3.9% to 22.5% of the participants reported early resumption of sexual activity [14]. Studies with less rigorous implementation protocols conducted in Kenya, Zambia, and Tanzania found that between 25% and 50% of men engaged in sexual activity before the wound had healed [12, 15–18]. These data suggest that early resumption of sexual activity is common in real world settings and therefore may attenuate the potential benefits of VMMC [12, 19, 20].

Given the high prevalence of early resumption of sexual activity in non-trial settings, strategies are needed to address this challenge. Previously identified risk factors for early resumption of sex include older age, married or living with a partner, engaging in risky sexual behavior, and concerns about losing sexual partners [15, 16, 18]. However, limited evidence exists on effective strategies for encouraging post-procedure sexual abstinence; a recently published literature review highlighted the need for additional research on facilitating abstinence during wound healing [21]. A randomized controlled trial conducted among 1,200 circumcised adult men in Kenya evaluated the effect of providing a series of text messages during the post-operative period on self-reported resumption of sexual activity prior to six weeks. Although the intervention was unsuccessful at improving post-procedure abstinence, the authors were able to identify risk factors for early resumption of sexual activity. They also highlighted the need for in-depth qualitative studies to better understand the reasons for early resumption of sex [16], as have other studies [22]. To maximize safety and ensure the population-level benefits of VMMC against HIV infection in real world settings, abstinence needs to be effectively promoted during the six-week period following VMMC. Using qualitative methods, we sought to better understand early resumption of sex following VMMC in Botswana.

## Methods

The study was conducted by the International Training and Education Center for Health (I-TECH), which is a collaboration between the University of Washington and University of California, San Francisco. Ethical approvals for the study were received from the Health Research and Development Committee at the Botswana Ministry of Health as well as the University of Washington Institutional Review Board. All procedures performed in studies involving human participants were in accordance with the ethical standards of the institution and/or national research committee and with the 1964 Helsinki declaration and it later amendments or comparable ethical standards.

**Fig 1:**
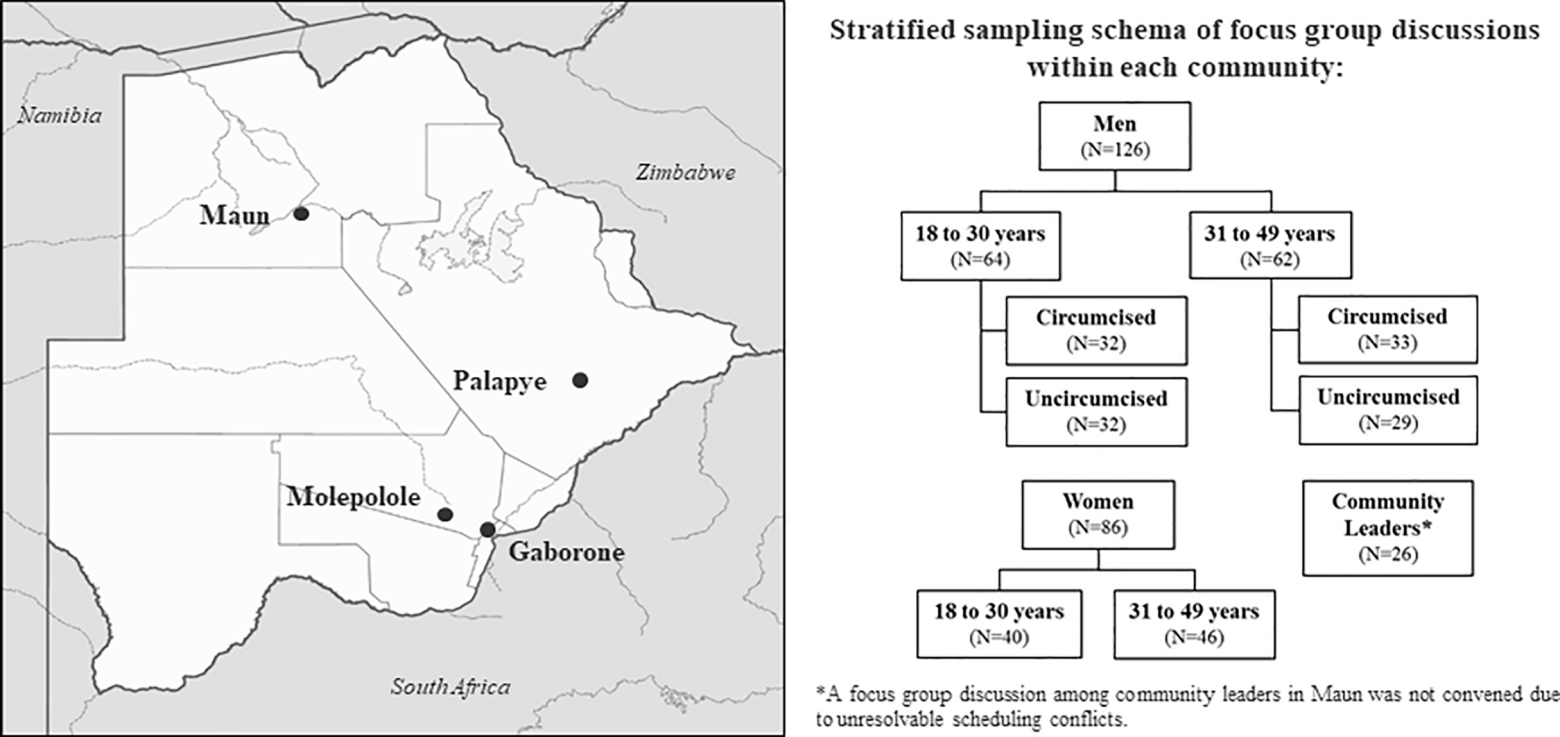
Map of selected communities and stratified sampling schema of focus discussion groups among men, women and community leaders according to age and circumcision status, Botswana, July to November 2013.

Focus group discussions were conducted among adult men, women, and community leaders in four communities in Botswana: Gaborone, Palapye, Molepolole, and Maun. These sites were purposefully selected based on district, predominant ethnic affiliation, and volume of circumcisions performed within the community. Specifically, the chosen communities are located in different districts within Botswana and primarily serve populations with different ethnic affiliations. Two communities with high-volume VMMC and two communities with low-volume VMMC clinics were chosen to represent VMMC clinics operating in the top 25% and bottom 25% of the total number of circumcisions performed nationwide, respectively. The two communities supporting high-volume VMMC clinics included Gaborone, the capital city of Botswana, and Palapye, a village 240 km northeast of Gaborone. The two communities supporting low-volume VMMC clinics included Molepolole, a village 50 km northwest of Gaborone, and Maun, the administrative center of a rural district located in the northwest region of Botswana (Fig 1).

Participants were eligible for participation if they were aged 18 to 49 years and willing to provide written informed consent and to be audio-recorded. No upper age limit was applied to community leaders. Within each community, convenience sampling was used to recruit subjects according to age and circumcision status (males only). Study staff approached and screened potential participants at the central bus station and shopping malls within the community. Two focus group discussions were convened each among circumcised men, uncircumcised men, and women with one group in each category reserved for individuals aged 18 to 30 years and one among those aged 31 to 49 years (Fig 1). Circumcision status for male participants was based on self-report. The number of participants in each focus group ranged from 5 to 14 individuals. Community leaders were recruited through the offices of local political, religious, and educational leaders. Each office or organization was sent a letter introducing the study and inviting their participation, followed by an in-person meeting or phone call to confirm interest in and availability for participation. All discussions were carried out at mutually-agreeable times and places, typically in the evening, to maximize participation. A focus group discussion planned among community leaders was not held in Maun due to unresolvable scheduling conflicts among participants.

A trained same-sex interviewer facilitated the discussions in the local language, Setswana, using a semi-structured discussion guide (S1 and S2 Texts). This included open-ended questions related to early resumption of sex followed by probing questions generated a priori based on the published literature. All discussions were audio-recorded, transcribed verbatim, and translated to English. Written informed consent for participation in and recording of the discussion were obtained for all participants. Basic demographic information was collected from each participant and directly entered into a customized REDCap (Research Electronic Data Capture) database hosted at the University of Washington [23].

Following the independent content analysis method, we began by having two investigators (KW, JL) individually identify concepts and key themes suggestive of early resumption of sex after reviewing all transcripts. The concept lists generated by each investigator were shared and discussed to construct a coding scheme consisting of a unified list of concepts and standardized definitions for each concept. Two members of the research team (KW, NR) independently coded the same randomly selected transcript to assess consistency between coders. Areas of inconsistency were discussed, noting why a particular code was assigned, and if appropriate, the operational definition was modified. The entire dataset was then reviewed and coded by both team members using Atlas.ti v7.0 software (http://www.atlasti.com). Any discrepancies on the assignment of concept codes were resolved by a third team member (JL). The coded data was then used to develop thematic categories that correspond to and represent early resumption of sex and risk compensation identified through the analysis. Categories were developed by grouping codes and coded data into meaningful clusters to form larger concepts. The final version of each category contained the category label or name, the operational definition of the category, and examples of the data from which the category was constructed.

## Results

A total of 238 men, women, and community leaders participated in 27 focus group discussions from July to November 2013 (S1 Table). Table 1 summarizes the demographic characteristics of the participants. Median age was 31 years, and 60% were male. Thirty-four participants (14%) reported a primary education or less while 122 (51%) reported at least a secondary education. Only 12% of participants were married.

**Table 1:** Descriptive characteristics of 238 men, women, and community leaders who participated in 27 focus group discussions across four communities in Botswana.

		N	(%)^a^
Location			
	Gaborone	69	(29%)
	Maun	51	(21%)
	Molepolole	58	(24%)
	Palapye	60	(25%)
Age, years, median (IQR)^b^		31	(24, 38)
Male gender		143	(60%)
Highest level of education completed			
	Primary or less	34	(14%)
	Secondary	122	(51%)
	Certificate level	42	(18%)
	Diploma	30	(13%)
	Degree	10	(4%)
Current relationship status			
	Never married	78	(33%)
	Dating and living together	42	(18%)
	Dating, but not living together	79	(33%)
	Married	29	(12%)
	Divorced, separated or widowed	10	(4%)
Religious affiliation			
	Christianity	207	(87%)
	Badimo^c^	9	(4%)
	No religious affiliation	17	(7%)
	Other	5	(2%)
Employment status			
	Employed and receiving a salary	64	(27%)
	Self-employed	67	(28%)
	Unemployed	76	(31%)
	Student	31	(13%)
Occupation			
	Professional or managerial	41	(31%)
	Skilled worker	48	(36%)
	Unskilled worker	41	(31%)
	Other	2	(2%)

IQR, interquartile range

^a^Percentages may not sum to 100% due to rounding.

^b^25^th^ and 75^th^ percentiles

^c^Traditionally circumcising ethic group

### Painful sex as a deterrent to early resumption of sexual activity

Pain, not HIV risk, was perceived as the main adverse consequence of early resumption of sex. Pain was reported by participants across all focus groups as the main adverse outcome of early resumption of sex post-circumcision (Table 2; Row 1–4). No participant mentioned a potential increase in HIV infection risk associated with early resumption of sex. Despite acknowledging pain as an undesirable consequence of engaging in sex prior to complete wound healing, it did not necessarily prevent early initiation of sex. One circumcised man stated, “I blame us who have circumcised because we keep telling them [uncircumcised men] that it [sex] is painful and it tears the sutures. But if you feel the pain, just get off the bed, put your feet on the floor, and the coldness from the floor will make you forget about the pain” (Table 2; Row 3).

**Table 2:** Early resumption sex following voluntary medical male circumcision (VMMC) from the perspectives of 238 men, women, and community leaders in four communities in Botswana.

Row	Theme	Focus Group Discussion	Representative quotation
1	Painful sex as a deterrent to early resumption of sexual activity	Women, 31 to 49 years, Gaborone	It is a difficult situation…the problem is that at an older age, men think a lot about sex. After circumcision, before the wound has fully recovered, men are likely to have strong desires to have sex with their partners. This results in them going through much pain.
2		Uncircumcised men, 31 to 49 years, Molepolole	As a guy, I believe you know that you can’t survive that long without wanting to do a little something [sex]. Now imagine when you get an erection and you have those stitches, I just can’t imagine the pain and like I said, six weeks of no sex is torture.
3		Circumcised men, 31 to 49 years, Palapye	If you were able to withstand the pain for an hour, that is what I call initiation. If you were able to withstand that pain in the morning after sex tore the sutures apart, you are a real man. We men are loose in conduct…we fail to just abstain for six weeks. I blame us who have circumcised because we keep telling them [uncircumcised men] that it [sex] is painful and it tears the sutures; but if you feel the pain, just get off the bed, put your feet on the floor, and the coldness from the floor will make you forget about the pain.
4		Circumcised men, 18 to 30 years, Molepolole	In some instances, I have seen guys who tried to have sex after two weeks and they were forced to go back to the hospital
5	Demonstration of masculinity and virility	Uncircumcised men, 31 to 49 years, Gaborone	As men our behaviors are diverse, some men cannot imagine six weeks of not engaging in sexual activities. I feel if the wound took a shorter period of time to heal, things would be better.
6		Circumcised men, 31 to 49 years, Maun	Some are impatient or should I say they are addicted. Sex is very addictive in the sense that if you are used to having sex regularly some men become anxious at that thought [of abstaining for six weeks], because there is a belief that if you go for long without sex you may go crazy … they say the ‘sack’ [testicles] will drive you crazy!
7		Uncircumcised men, 18 to 30 years, Palapye	The other issue is six weeks. It is a long time and men wonder whether they can afford to stay for that long without doing anything sexual because the nerves or veins may block in one way or the other.
8		Circumcised men, 18 to 30 years, Palapye	There are men with high libido and high sex drive, who want to have sex every day. They think they are strengthening their bodies. If they think of going for such a long time without doing anything sexually, they cringe.
9	Fear of losing female partners	Circumcised men, 31 to 49 years, Palapye	When we think of circumcision most of us start having thoughts of other men taking our women whilst we are recuperating. The woman will also feel sexually starved, and I will think that so-and-so [another man] will take my girl because he has being following her and he may have even encouraged me to circumcise.
10		Circumcised men, 18 to 30 years, Maun	If my friend goes to circumcise, I will be surprised and say to him that “you have gone to circumcise and I am going to sleep with your girl”. That is one of the issues that scares men, these six weeks. There is a high possibility of losing a girl in that six weeks. Men are afraid that they [other men] will take their women.
11		Women, 31 to 49 years, Gaborone	Men may think that women are likely to cheat on them whilst they are still in the six weeks healing period.
12		Circumcised men, 18 to 30 years, Palapye	During the recuperation period, woman may go and look for something [sex] somewhere else because of loneliness.
13	Misperceptions about post-operative wound healing	Women, 18 to 30 years, Gaborone	It does not even take long to heal. One of my friends took just two weeks or even less to heal.
14		Women, 18 to 30 years, Gaborone	You do not take the whole month with the wound…it takes about two weeks to heal. I really do not understand what they are scared of, basically yes it is painful, but after two weeks I think they can assume sex.
15		Women, 31 to 49 years, Maun	Men are worried about the time they are given before they can have sex, if I am not mistaken it’s a month.
16		Uncircumcised men, 31 to 49 years, Maun	From what I have observed and heard, when we are discussing these things as men, we only focus on negatives like it is painful, things like you are going to spend three weeks without having sex and stuff like that. Those are the main things that we talk about.
17	Influence of women to promote post-operative abstinence	Circumcised men, 31 to 49 years, Gaborone	I told her that I was instructed from the hospital not to be engaged in any sexual activities and should stay [abstinent] for a specific period.
18		Women, 31 to 49 years, Palapye	I have been with my partner for six years. In 2010 he went to be circumcised. He never gave me any troubles though at times before he could heal he would attempt to lure me in to having sex with him and I would tell him to take it easy as he is yet to heal.
19		Women, 31 to 49 years, Gaborone	I think after circumcision, if you stay together with your partner, there should be a certain way you care for him. You sleep in separate beds to avoid contact…if there is contact there would be erection, hence more pain caused by the sutures tightening
20		Women, 31 to 49 years, Palapye	Yes, you will hear someone saying ‘Hey I am so horny’ then you just have to tell him to persevere until he has recovered
21		Women, 31 to 49 years, Maun	When I met my current man, I asked him to go with me for testing. When we got there they encouraged him to get circumcised. We discussed the issue and he did not refuse and he went to get circumcised. I used to visit him to check on him…I never spent a night at his place until the wound was completely healed. He is now fine.
22		Women, 18 to 30 years, Palapye	When you are living together in the same house it is difficult…I put a sanitary pad on and told him that I was having my period.
23	Linking VMMC to the post-partum period to promote post-operative abstinence	Circumcised men, 18 to 30 years, Gaborone	It might be easier if you get your partner pregnant and then while on confinement, you get circumcised so that the both of you can wait six weeks.
24		Molepolole Leaders	I look at this issue and think of confinement. When my wife had her first child, I had to stay away from her for three months without having sex and I believe that is what most men have experienced. My question now is, if that was possible, what makes us now think that 6 weeks is a long time. All I can say is men are just fronting their lustful desires instead of being worried about their health
25		Women, 31 to 49 years, Palapye	I have a younger brother who told me that six weeks is a long time, but I reminded him that he has children and much as he was able to wait for his woman during confinement he should do likewise about circumcision.
26	Infant male circumcision to eliminate post-operative abstinence concerns	Women, 18 to 30 years, Molepolole	Maybe the reason they do not circumcise when they are older is because they feel the six weeks they have to wait before resuming sex is long; because they are reluctant to do it when they are older, let’s do it at birth.
27		Circumcised men, 31–49 years, Palapye	When you are older circumcision is a daunting task…it is best if they are circumcised when they are young because then they are still innocent.
28		Women; 18 to 30 years, Palapye	I suggest that they get circumcised at a younger age because if they are already sexually active or living with a girlfriend; it won’t be easy to abstain during the recuperating time. I think it will be best to circumcise them when they are still young.

### Demonstration of masculinity and virility

Demonstrating masculinity and virility was a primary motivator for resuming sexual activity during the six-week abstinence period. Participants across all discussion groups, including those convened with women, felt that abstaining from sex was a challenge. Sex was described as “addictive” and refraining from sexual activity for even short periods of time was “torture” (Table 2; Rows 6 and 2, respectively). Many participants stated that it was abnormal for men to abstain from sex for six weeks. Furthermore, male participants in particular indicated that sex was important for maintaining good health (Table 2; Rows 6–8).

### Fear of losing female partners

In addition to the virility of male partners, virility of female partners was also seen as a barrier to adherence to the post-operative abstinence period (Table 2; Row 9–12). Focus group participants commonly mentioned that potential promiscuity among female partners was a concern. As stated by a circumcised older man in Palapye, “When we think of circumcision most of us start having thoughts of other men taking our women whilst we are recuperating. The woman will also feel sexually starved. That is one of the issues that scare men, these six weeks” (Table 2; Row 9). This was succinctly described by a circumcised participant from Maun who stated that “There is a high possibility of losing a girl in that six weeks” (Table 2; Row 11).

### Misperceptions about post-operative wound healing

In several discussion groups, there were clear misperceptions related to the recommended time period of abstinence post-circumcision. In some cases, female participants suggested the healing period was shorter than six weeks (Table 2; Rows 13–15). One woman stated, “It does not even take long to heal. One of my friends took just two weeks or even less to heal.” (Table 2; Row 13) While misperceptions related to the duration of the abstinence period were more common within the focus groups with women, misperceptions were also mentioned in discussions with men (Table 2; Row 16).

### Influence of women to promote post-operative abstinence

Both male and female participants volunteered strategies on how women could encourage abstinence during the wound healing period. One older circumcised man recounted his own experience with his partner following VMMC, indicating that he purposefully communicated with his partner that “[he] was instructed from the hospital not to be engaged in any sexual activities.” (Table 2; Row 17). This experience, however, was not commonly reported by other male participants. In contrast, women were more likely to identify a role in encouraging abstinence during the post-operative period (Table 2; Rows 18–22). This included reminding their partners and/or male relatives of the need for abstinence (Table 2; Row 18) and undertaking strategies targeted at reducing sexual desire, including sleeping in separate beds during the wound healing period.

### Linking VMMC to the post-partum period to promote post-operative abstinence

Participants frequently compared the abstinence period following VMMC with the postpartum abstinence undertaken by women (Table 2; Row 23–25). One male participant explicitly suggested that VMMC be linked to the postpartum period, stating that “It might be easier if you get your partner pregnant and then while on confinement [postpartum abstinence period], you get circumcised so that both of you can wait six weeks” (Table 2; Row 23). Similarly, participants felt circumcision-related abstinence should be treated no differently than abstinence related to the postpartum period. One community leader stated the following: “I look at this issue and think of confinement. When my wife had her first child, I had to stay away from her for three months without having sex and I believe that is what most men have experienced. My question now is, if that was possible, what makes us now think that six weeks is a long time.” (Table 2; Row 24).

### Infant male circumcision to eliminate post-operative abstinence concerns

Many participants indicated that the challenges related to post-operative sexual abstinence could be solved by promoting medical male circumcision among male infants and children (Table 2; Rows 26–28). Circumcising infants and boys prior to sexual debut would eliminate any challenges related to early resumption of sex. One female participant stated, “I suggest that they get circumcised at a younger age because if they are already sexually active or living with a girlfriend it won’t be easy to abstain during the recuperating time. I think it will be best to circumcise them when they are still young” (Table 2; Row 28).

## Discussion

This study highlights a potentially widespread lack of awareness of the increased risk of HIV acquisition during the wound healing period. Pain, not risk of HIV acquisition, was perceived as the main deterrent of early resumption of sex. This adverse consequence was not, however, sufficient for maintaining abstinence during wound healing. Participants strongly believed that regular sex was not only normal but required for maintaining health and mitigating partner infidelity. Strategies to promote post-procedure abstinence shared by participants included educating women about the wound healing process, linking VMMC with female partners’ post-partum period, and encouraging infant male circumcision.

Previously published data have demonstrated that sex during post-circumcision wound healing is not only common [12, 15–18] but may also increase the risk of major adverse events including HIV acquisition [11–14, 19, 20]. Mathematical modeling has suggested that early resumption of sex would not likely outweigh the population-level benefits, given the strength of the protective benefit of VMMC after the circumcision wound has healed [12, 14, 19, 20]. It is, nevertheless, critical that VMMC services are delivered in a manner that ensures the safety of clients and their partners. There is a clear need to ensure that health workers highlight that early resumption of sex puts both clients and their partners at heightened risk of HIV infection.

As VMMC programs grow, they will need to increase their focus on older men to reach overall program targets, which will make finding strategies to motivate post-procedure abstinence critical. Three studies from Kenya have found that older men are more likely to resume sex prior to wound healing compared to younger men [15, 16, 18]. For example, one study found that among men who resumed sex prior to six weeks, 25% were 30 years or older whereas only 8% of those who adhered to the abstinence guideline were in this age group [15]. Strong post-procedure counseling for older men will also be important because HIV incidence and prevalence are highest in these populations [24–26]. In the case of Botswana, HIV incidence and prevalence peaks among men aged 45–49 years [27] and 40–44 years, respectively [28].

Increased focus on implementing effective strategies to support post-procedure abstinence will be critical among not only older men but also HIV-positive clients undergoing VMMC. Resumption of sexual activity by HIV-positive clients prior to complete wound healing would potentially increase risk of transmission to HIV-negative partners. HIV infection is not a contraindication for VMMC. Moreover, there are non-HIV-related medical benefits, including improved hygiene, reduced STI risk and reduced genital cancers in men and their female sexual partners [29, 30]. Yet, data from Uganda suggest that this population may be more likely than HIV-negative men to resume sex before complete wound healing [11].

Several recommendations can be considered for promoting sexual abstinence following VMMC based on data from this qualitative study. First, men undergoing circumcision, together with their partners, may benefit from additional counseling on the temporary increase in HIV risk during wound healing. Second, new and innovative social messaging should be developed to promote post-VMMC abstinence as a healthy social norm and to encourage women to support their partners during this period. Involving women may be particularly important. Reports from Kenya found that men resuming sexual activity early were more likely to report that women were increasingly attracted to them after VMMC [15]. Another strategy to encourage adherence to post-procedure abstinence was promoting linkages between VMMC, antenatal care and post-partum services. Healthcare workers in antenatal clinics are well-positioned to promote VMMC services to partners of pregnant women to align the timing of the post-procedure abstinence period for VMMC with the abstinence period already observed after labor and delivery.

A strength of this study was that a variety of individuals were purposefully sampled according to community, age, gender, and circumcision status. Our participants were recruited from communities varying prevalence of HIV infection and VMMC; at the time of data collection, HIV prevalence among males of all ages ranged from 13% in Maun to 20% in Molepolole (compared to 16% across the entire country). VMMC prevalence also varied, albeit to a smaller degree, across study communities; in 2013 approximately 22% of men aged 10 to 64 years in Palapye were circumcised compared to 26% in Gaborone and 25% nationwide [30]. The study encompassed 238 men and women as well as community leaders to ensure that a wide range of perspectives were represented. Nevertheless participants were recruited using convenience sampling, which may introduce bias. In addition, circumcision status was assessed by self-report, which has been shown to not always be accurate [31]. Participants may, however, have been inhibited to speak freely due to the traditional cultural and social norms related to discussing sexual behavior. Skilled, same sex moderators were used to mitigate this limitation.

Evidence suggests that men and women are not well informed about the increase in HIV risk during wound healing. As VMMC programs in sub-Saharan Africa aggressively move forward to reach population-level scale-up targets, greater numbers of older men as well as men who are HIV-positive will seek VMMC services. Attention to early resumption of sex following VMMC will be increasingly important, especially given that to date, clients and their partners appear unaware of the increased risk of HIV prior to complete wound healing and effective strategies to promote abstinence following VMMC are lacking [16].

## Supporting information

S1 Text Focus group discussion tool in English. (DOCX)

S2 Text Focus group discussion tool in Setswana. (DOC)

S1 Table Demographic data file of focus group participants. (CSV)
