# Supplementary material for: Early resumption of sexual activity following voluntary medical male circumcision in Botswana: A qualitative study
Source: PLoS One. 2017 Nov 14;12(11):e0186831. doi: 10.1371/journal.pone.0186831 (PMC5685600; doi:10.1371/journal.pone.0186831)
Supplement: S1 Text — (DOCX) [file pone.0186831.s001.docx]

**Appendix K: Focus Group Discussion Guide**

**Topical areas: Attitudes and beliefs about circumcision, sexual norms post-circumcision campaign, reasons to circumcise, and reasons not to circumcise**

Thank you for your participation in our focus group discussion on safe male circumcision. My name is ________________ and I will be moderating the discussion. I anticipate the discussion will last about 60 minutes, and I appreciate any information you can provide. Your participation in the discussion is strictly voluntary, and you may withdraw at any time, and for any reason.

These focus groups are important for the success of evaluating the National SMC Program, and will serve to increase our understanding of the impact of SMC on the community. In total, 28 focus group discussions will be held in 4 different communities in Botswana. Focus groups will be made up of men of different ages and circumcision status, women of different ages, and community leaders.

We will ask questions about your perceptions of male circumcision, factors affecting why men get circumcised, and the influence of SMC on sexual behaviors. Any information you give us will be kept private and will be recorded without using names. Although your responses will be reported as part of a group, the recording of these responses will help ensure accuracy of the reports. We ask that you do not share what is said today with anyone outside the group. I will now ask each participant the following questions and your answer will be recorded on tape. Please answer “yes” or “no”. Do you give your consent to participate in this focus group discussion? Is it okay if I audio-record this interview?

**MEN: CIRCUMCISED/UNCIRCUMCISED**

**Warm-up**

What have you heard about adult male circumcision?

PROBE: Relationship between circumcision and HIV?

Relationship between circumcision and sexually transmitted infections?

Benefits of circumcision for women?

**Attitudes, Beliefs, and Perceptions about SMC**

What is the best age for a man to be circumcised?

PROBE: Why?

How does age influence whether or not a man is circumcised?

Would men your age be more interested in getting circumcised if they were offered it at a

younger age?

How is adult male circumcision viewed in your community?

PROBE: What do men say when they talk about the circumcision procedure with each other?

If a friend or family member said that he is circumcised, how would that make you feel?

How would other members of your community react when hearing that a man is circumcised?

What are men most worried about before circumcision?

PROBE: The surgery itself?

Potential complications or negative side effects of the procedure?

How do these worries affect a man’s decision to get circumcised?

Do you think women prefer circumcised partners? Why or why not?

**Changes in Sexual Behavior**

How have men in your community changed their sexual behavior because of circumcision?

PROBE: Are they less likely to use condoms? More likely? Why?

Do circumcised men believe it okay to expect sex without a condom because he is circumcised?

How have men’s reactions changed when their partner asks them to use a condom?

How have relationships (casual, dating, or marital relationships) changed because of circumcision?

PROBE: Are men more likely to have multiple sexual partners? Less likely?

Are men more likely to have multiple sexual partners at the same time? Less likely?

Are sexual partners more or less open to discussing HIV status and safe sex practices with each other?

**Factors Influencing Uptake of SMC**

- **Reasons to Circumcise (facilitators)**

Why do men your age choose to get circumcised?

PROBE: Protection against HIV infection?

Partner wants man to be circumcised?

Recommendation from family or friends?

Circumcision is supported by the community?

Hygiene?

Prefer appearance of circumcised penis?

- **Reasons not to Circumcise (barriers)**

Why do men your age choose not get circumcised?

PROBE: Do not know circumcision is available or benefits of circumcision?

Not able to take time off from work?

Cannot afford transport costs to get to and from clinic?

Scared of pain during and after surgery?

Scared surgery will lead to decreased sensation or pleasure during intercourse?

Length of healing time?

Before a man is circumcised as an adult, he must receive an HIV test. How does mandatory HIV testing prior to getting circumcised affect a man’s decision to get circumcised?

PROBE: Are men scared to learn their status?

Are men worried that other people will find out their HIV status?

If you wanted to get circumcised and you are employed, what would your employer say?

PROBE: If a man could be circumcised and return to work the same day as their circumcision, how would

this change the way men felt about circumcision? Would more men get circumcised?

If there was a pain-free option for circumcision that did not involve surgery, how would this change the way men felt about circumcision? Would more men get circumcised?

If a man wants to get circumcised, how would he know where to go?

PROBE: Tell me about the circumcision campaign in your community. What seems to work well (or not at

all) as part of the campaign to increase the number of adult men getting circumcised?

Additional PROBE: Television commercials?

Radio advertisements?

Home visits by a community member?

**If men admit to being circumcised, then ask this question:**

If you could go back and make the decision again, would you still decide to get circumcised?

PROBE: Why or why not?

Would you recommend circumcision to your male family members or friends?

What are some benefits, if any, of being circumcised that you did not expect? Any negative effects?

**Closing:**

Is there anything else you would like to say before we end our discussion? Thank you for agreeing to participate in this focus group discussion. Your input will assist the nation in the fight against HIV and AIDS, and it will contribute to our understanding of the impact of male circumcision and how we can improve SMC services in Botswana.

**WOMEN**

**Warm-up**

What have you heard about adult male circumcision?

PROBE: Relationship between circumcision and HIV?

Relationship between circumcision and sexually transmitted infections?

Benefits of circumcision for women?

**Attitudes, Beliefs, and Perceptions About SMC**

What is the best age for a man to be circumcised?

PROBE: Why?

How does age influence whether or not a man is circumcised?

Would men your age be more interested in getting circumcised if they were offered it at a

younger age?

How is adult male circumcision viewed in your community?

PROBE: What do men say when they talk about the circumcision procedure with each other?

What do women say?

How do members of your community react when hearing that a man is circumcised?

What are men most worried about before circumcision?

PROBE: The surgery itself?

Potential complications or negative side effects of the procedure?

How do these worries affect a man’s decision to get circumcised?

Do you know anyone, including yourself, who had a partner get circumcised in the last 3 years?

PROBE: How did they (or you) feel when their partner said they wanted to be circumcised?

Happy with their decision?

Worried it would change the relationship?

Worried about possible surgical complications?

Do you know anyone, including yourself, who asked their partners to get circumcised?

PROBE: How did the men react?

What did they say?

Do you think women prefer circumcised partners? Why or why not?

PROBE: If their partner is circumcised, do women feel safer not using a condom during sex?

**Changes in Sexual Behavior**

How have men in your community changed their sexual behavior because of circumcision?

PROBE: Are they less likely to use condoms? More likely? Why?

Do circumcised men believe it is okay to expect sex without a condom because he is circumcised?

What do they say to their female partner when they don’t want to use a condom?

Are women your age comfortable asking their circumcised partner to wear? What if

Their partner was not circumcised?

How have relationships (casual, dating, or marital relationships) changed because of circumcision?

PROBE: Are men more likely to have multiple sexual partners? Less likely?

Are men more likely to have multiple sexual partners at the same time? Less likely?

Are sexual partners more or less open to discussing HIV status and safe sex practices with each other?

**Factors Influencing Uptake of SMC**

- **Reasons to Circumcise (facilitators)**

Why do men your age choose to get circumcised?

PROBE: Protection against HIV infection?

Partner wants man to be circumcised?

Recommendation from family or friends?

Circumcision is supported by the community?

Hygiene?

Prefer appearance of circumcised penis?

- **Reasons not to Circumcise (barriers)**

Why do men your age choose not get circumcised?

PROBE: Do not know circumcision is available or benefits of circumcision?

Not able to take time off from work?

Cannot afford transport costs to get to and from clinic?

Scared of pain during and after surgery?

Scared surgery will lead to decreased sensation or pleasure during intercourse?

Length of healing time?

What affect do women have on encouraging men to get circumcised?

PROBE: How can women help increase the number of circumcised men?

If a man wants to get circumcised, how would he know where to go?

PROBE: Tell me about the circumcision campaign in your community. What seems to work well (or not at

all) as part of the campaign to increase the number of adult men getting circumcised?

Additional PROBE: Television commercials?

Radio advertisements?

Home visits by a community member?

**Closing:**

Is there anything else you would like to say before we end our discussion? Thank you for agreeing to participate in this focus group discussion. Your input will assist the nation in the fight against HIV and AIDS, and it will contribute to our understanding of the impact of male circumcision and how we can improve SMC services in Botswana.

**COMMUNITY LEADERS**

**Warm-up**

What have you heard about adult male circumcision?

PROBE: Relationship between circumcision and HIV?

Relationship between circumcision and sexually transmitted infections?

Benefits of circumcision for women?

**Attitudes, Beliefs, and Perceptions About SMC**

What is the best age for a man to be circumcised?

PROBE: Why?

How does age influence whether or not a man is circumcised?

Would men be more interested in getting circumcised if they were offered it at a

younger age?

How is adult male circumcision viewed in your community?

PROBE: What do men say when they talk about the circumcision procedure with each other?

What do women say?

How do members of your community react when hearing that a man is circumcised?

What are men most worried about before circumcision?

PROBE: The surgery itself?

Potential complications or negative side effects of the procedure?

How do these worries affect a man’s decision to get circumcised?

**Changes in Sexual Behavior**

How have men in your community changed their sexual behavior because of circumcision?

PROBE: Are they less likely to use condoms? More likely? Why?

Do circumcised men believe it is okay to expect sex without a condom because he is circumcised?

What do they say to their female partner when they don’t want to use a condom?

Are women your age comfortable asking their circumcised partner to wear? What if

Their partner was not circumcised?

How have relationships (casual, dating, or marital relationships) changed because of circumcision?

PROBE: Are men more likely to have multiple sexual partners? Less likely?

Are men more likely to have multiple sexual partners at the same time? Less likely?

Are sexual partners more or less open to discussing HIV status and safe sex practices with each other?

**Factors Influencing Uptake of SMC**

- **Reasons to Circumcise (facilitators)**

Why do men your age choose to get circumcised?

PROBE: Protection against HIV infection?

Partner wants man to be circumcised?

Recommendation from family or friends?

Circumcision is supported by the community?

Hygiene?

Prefer appearance of circumcised penis?

- **Reasons not to Circumcise (barriers)**

Why do men your age choose not get circumcised?

PROBE: Do not know circumcision is available or benefits of circumcision?

Not able to take time off from work?

Cannot afford transport costs to get to and from clinic?

Scared of pain during and after surgery?

Scared surgery will lead to decreased sensation or pleasure during intercourse?

Length of healing time?

What affect do women have on encouraging men to get circumcised?

PROBE: How can women help increase the number of circumcised men?

We know that circumcision rates among older men are low. Why do you think older men (over 30) are not

getting circumcised?

PROBE: How can leaders in the community help to increase the number of men getting circumcised?

If a man wants to get circumcised, how would he know where to go?

PROBE: Tell me about the circumcision campaign in your community. What seems to work well (or not at

all) as part of the campaign to increase the number of adult men getting circumcised?

Additional PROBE: Television commercials?

Radio advertisements?

Home visits by a community member?

**Closing:**

Is there anything else you would like to say before we end our discussion? Thank you for agreeing to participate in this focus group discussion. Your input will assist the nation in the fight against HIV and AIDS, and it will contribute to our understanding of the impact of male circumcision and how we can improve SMC services in Botswana.
