# Supplementary material for: Early resumption of sexual activity following voluntary medical male circumcision in Botswana: A qualitative study
Source: PLoS One. 2017 Nov 14;12(11):e0186831. doi: 10.1371/journal.pone.0186831 (PMC5685600; doi:10.1371/journal.pone.0186831)
Supplement: S2 Text — (DOC) [file pone.0186831.s002.doc]

**Appendix K: Kaedi ya Potostso ka setlhothswana**

**Ditlhogo: Maikutlo le ditumelo ka loaro la bonna, melawana ya tlhakanelo dikobo morago ga dithuto-phatlatsa loaro la bonna, mabaka a go dira loaro la bonna, le mabaka a go tlhoka go dira loaro la bonna**

Re lebogela go tsaya karolo ga gago mo puisanyong ya rona ya go dira loaro la bonna lo lo babalesegileng. Leina la me ke ______________________, ke nna ke tla a bong ke tsamaisa puisanyo e. Ke solofela gore puisanyo e tla a tsaya lobaka la metsotso e le masome a marataro, mme ke lebogela dikgang tsotlhe tse lo tla a di re neelang. Go tsenelela ga gago mo puisanyong e ga go patelediwe, ebile o letlelelwa go ikgogela morago ka nako epe fela le ka mabaka ape fela.

Dipuisanyo ka ditlhotshwana tse di botlhokwa mo go sekasekeng lenaneo la sechaba la loaro la banna lo lo babalesegileng ebile di tla godisa ka fa re tlhaloganyang ka teng boleng mo morafeng. Ka kakaretso, re tla a buisanya le ditlhotshwana di le masome a mabedi le boferabobedi mo merafeng e e farologanyeng mo Botswana. Ditlhotshwana di tla a dirwa ka borre ba dingwaga tse di farologanyeng, ba ba dirileng loaro la bonna le ba ba sa le dirang, bomme ba dingwaga tse di farologanyeng, le bagogi ba merafe.

Re tla a lo botsa dipotso ka dikakanyo tsa lona ka loaro la bonna, tse di rotletsang borre go dira loaro la bonna, le ka fa loaro la bonna le amang ka teng maitshwaro a tlhakanelo dikobo. Dipe fela dikgang/dintlha tse lo di re neelang re tla a di babalela ebile di tla a gatisiwa mo sekapa-mantsweng go sa dirisiwe maina. Le fa dikarabo tsa lona di tla a begwa e le bontlha bongwe jwa setlhopha, go gatisa dikarabo tse ka sekapa-mantswe go tla a re thusa go netefatsa boammaruri jwa dipego. Re kopa gore o seke wa amogana se re se buileng gompieno le ope fela yo o seng mo setlhopheng se. Jaanong ke tla a kopa mongwe le mongwe wa batsayakarolo dipotso tse di latelang mme dikarabo tsa lona di tla a gatisiwa ka sekapa-mantswe. Tswee-tswee araba “ee” kgotsa “nnyaa”. A o re fa teseletso ya go tsaya karolo mo puisanyong e? A o siame le gore re ka gatisa puisanyo e ka sekapa-mantswe?

**BANNA: BA BA DIRILENG LOARO LA BONNA/BA BA SA DIRANG LOARO LA BANNA**

**Ithokotso (warm-up)**

O utlwile eng ka loaro la banna lo lo diriwang mo bagolong?

GWETLHA: Kgolagano ya loaro la bonna le HIV?

Kgolagano ya loaro la bonna le malwetse a tlhakanelo dikobo?

Madup a loaro la bonna mo go bo mme?

**Maikutlo, Ditumelo, le Dikakanyo ka loaro la bonna**

Ke dingwaga dife tse di siametseng gore monna o ka dira loaro la bonna?

GWETLHA:

Ka go reng?

Dingwaga di ama jang gore a rre o dirile kgotsa ga a dira loaro la bonna?

A borre ba ba lekanang le wena ka dingwaga ba ka kgatlhegela go dira loaro la bonna fa ba ne ba ka rotloediwa go le dira ba le bannye?

Loaro la bonna le tsewa/lebiwa jang mo morafeng wa lona?

GWETLHA: Borre ba a reng fa ba buwa ka loaro la bonna le borre ba bangwe?

Fa tsala kgotsa mongwe wa losika a ka re o dirile loaro la bonna, o ka tseega jang?

Batho ba mo motseng ba dira jang fa ba ka utlwa gore rre/monna o dirile loaro la bonna?

Matshwenyego a borre a matona ke eng pele ga ba dira loaro la bonna?

GWETLHA: Loaro/go segwa?

Ditlamorago tse di sa siamang tse di ka nnang morago ga loaro?

Matshwenyego a, amang jang go tsaya tshwetso ga rre go dira loaro loaro la bonna?

A o akanya gore bomme ba kgatlhegela thata borre ba ba dirileng loaro la bonna? Ka goreng ba ba kgatlhegela kgotsa ka go reng ba sa ba kgatlhegele?

**Phetogo mo maitsholong a tlhakanelo dikobo**

Borre mo motseng wa lona ba fetotse jang maitshwaro a bone a tlhakanelo dikobo ka lebaka la loaro la bonna?

GWETLHA: A go lebega ba tla fokotsa tiriso ya sekausu? A ba tla oketsa tiriso ya sekausu? Ka go reng?

A borre ba ba dirileng loaro la bonna ba dumela gore go siame go amogela tlhakanelo dikobo go sa dirisiwe sekausu ka gore ba dirile loaro la bonna?

Go fetogile jang ka fa borre ba tseegang ka teng fa bakapelo ba bone ba ba kopa go dirisa sekausu?

Botsalano (jwa nakwana, go ratana, kgotsa manyalo) a fetogile jang ka lebaka la loaro la bonna?

GWETLHA:

A go lebega borre ba ka oketsa go nna le bakapelo ba ba farologanyeng? A go lebega ba ka fokotsa?

A go lebega borre ba ka oketsa go nna le bakapelo ba ba farologanyeng ka nako e le nngwe? A go lebega ba ka fokotsa?

A bakapelo ba gololesegile kgotsa ga ba a gololesega go buisana ka seemo sa mogare wa HIV le ditsela tse di babalesegileng tsa tlhakanelo dikobo?

**Tse di amang go dira loaro la bonna ke borre**

- **Mabaka a go dira loaro la bonna (tse di rotloetsang)**

Ke eng borre ba ba lekanang le wena ka dingwaga ba itlhophela go dira loaro la bonna?

GWETLHA: Go itshireletsa kgatlhanong le go fetelwa ke mogare wa HIV?

Mokapelo o batla gore rre a dire loaro la bonna?

Thotoetso go tswa mo go ba losika le ditsala?

Morafe o eme nokeng go dira loaro la bonna?

Bophepa?

Go kgatlhegela tebego ya bonna fa go dirilwe loaro la bonna?

- **Mabaka a go sa dira loaro la bonna (tse di kgoreletsang)**

Ke eng borre ba ba lekanang le wena ka dingwaga ba itlhophela go sa dira loaro la bonna?

GWETLHA:

Ga ba itse gore ditirelo tsa go dira loaro la bonna di teng kgotsa maduo a go dira loaro la bonna?

Ga ba kgone go bona nako go tswa ko tirong?

Ga ba kgone madi a dipalamo go ya le go bowa ko kokelong?

Ba tshaba botlhoko ka nako ya loaro le morago ga lone?

Ba tshoga gore loaro le ka tsala go fokotsega ga monate fa go tlhakanelwa dikobo?

Lebaka la go fola?

Pele ga rre a diriwa loaro la bonna fa e le mogolo, o tshwanetse go itlhatlhobela mogare wa HIV. Go pateletsa go itlhatlhobela mogare wa HIV pele ga go diriwa loaro la bonna go ama jang tshwetso ya rre go dira loaro la monna?

GWETLHA:

A borre ba tshaba go itse ka seemo sa bone sa mogare wa HIV?

A borre ba tshogela gore batho ba bangwe ba tla lemoga seemo sa bone sa mogare wa HIV?

Fa o batla go dira loaro la bonna mme o bereka, mohiri wa gago o ya go reng?

GWETLHA:

Fa rre a ka diriwa loaro la bonna a bo a boela ko tirong ka lone letsatsi le a dirilweng loaro ka lone, se se ka fetola jang tsela e borre ba ikutlwang ka yone ka loaro la bonna? A borre ba ka dira loaro la bonna ka bontsi?

Fa go na le tsela e nngwe ya go dira loaro la bonna e e seng botlhoko ebile go sa segiwe, se se ka fetola jang tsela e banna ba ikutlwang ka yone ka loaro la bonna? A borre ba ka dira loaro la bonna ka bontsi?

Fa rre a batla go dira loaro la bonna, o ya go itse jang gore o tshwanetse go ya kae?

GWETLHA: Mpolelela ka thuto-phatlatsa ya loaro la bonna mo motseng wa lona. Ke eng se se berekang sentle (kgotsa se se sa berekeng gotlhelele) e le bontlha bongwe jwa jwa thuto-phatlalatsa e e direlwang go oketsa dipalo tsa borre ba bagolo go diriwa loaro la bonna.

GWETLHA gape: ipapatso ya Television?

Ipapatso ya Radio?

Ketelo ko malapeng ka leloko la motse?

**Fa borre ba dumela go diriwa loaro la bonna, botsa dipotso tse:**

Fa o ne o ka boela ko morago o bo o dira tshwetso gape, a o ne o ka tswelela ka tshwetso ya go diriwa loaro la bonna?

PROBE: Ka gore kgotsa ka go reng o ka seke?

A o ka rotloetsa rre wa masika la gagokgotsa ditsala go dira loaro la bonna?

Ke afe maduo, fa a le teng, a go diriwa loaro la bonna a o neng o sa a solofela? A go na le ditlamorago tse di sa siamang?

**Bokhutlo:**

A go na le sengwe gape se o eletsang go se buwa pele ga re fetsa puisanyo ya rona? Ke go lebogela go dumela go tsaya karolo mo puisanyong e. Go tshwaela ga gago go tla a thusa sechaba mo ntweng kgatlhanong le mogare wa HIV le bolwetse jwa AIDS, ebile go tla nna le seabe mo go tlhaloganyeng se loaro la bonna lo ka se dirang le ka fa re ka toka fatsang ka teng lenaneo la loaro la bonna lo lo babalesegileng mo Botswana.

**BOMME**

**Ithokotso (warm-up)**

O utlwile eng ka loaro la banna lo lo diriwang mo bagolong?

GWETLHA: Kgolagano ya loaro la bonna le HIV?

Kgolagano ya loaro la bonna le malwetse a tlhakanelo dikobo?

Madup a loaro la bonna mo go bo mme?

**Maikutlo, Ditumelo, le Dikakanyo ka loaro la bonna**

Ke dingwaga dife tse di siametseng gore monna o ka dira loaro la bonna?

GWETLHA:

Ka go reng?

Dingwaga di ama jang gore a rre o dirile kgotsa ga a dira loaro la bonna?

A borre ba ba lekanang le wena ka dingwaga ba ka kgatlhegela go dira loaro la bonna fa ba ne ba ka rotloediwa go le dira ba le bannye?

Loaro la bonna le tsewa/lebiwa jang mo morafeng wa lona?

GWETLHA: Borre ba a reng fa ba buwa ka loaro la bonna le borre ba bangwe?

Bomme bone ba reng?

Batho ba mo motseng ba dira jang fa ba ka utlwa gore rre/monna o dirile loaro la bonna?

Matshwenyego a borre a matona ke eng pele ga ba dira loaro la bonna?

GWETLHA: Loaro/go segwa?

Ditlamorago tse di sa siamang tse di ka nnang morago ga loaro?

Matshwenyego a, amang jang go tsaya tshwetso ga rre go dira loaro loaro la bonna?

A o itse mongwe, o ipalela mo teng, yo o nang le mokapelo yo o dirileng loaro la bonna mo ngwageng tse tharo tse di fetileng?

GWETLHA:

O ne a (kgotsa o ne wa) ikutlwa jang fa mokapelo wa gagwe a re o batla go dira loaro la bonna?

A itumeletse tshwetso e?

A tshwenyegile gore go tla ama botsalano?

A tshogetse ditlamorago tse di ka nnang morago ga loaro?

A o itse mongwe, o ipalela mo teng, yo o kopileng mokapelo wa gagwe go dira loaro la bonna?

GWETLHA:

Borre ba bone ba ne ba ikutlwa jang?

Ba ne ba reng?

A o akanya gore a bomme ba kgatlhegela bakapelo ba ba dirileng loaro la bonna? Ka go reng kgotsa ka go reng ba sa ba kgatlhegele?

GWETLHA: Fa mokapelo a dirilwe loaro la bonna, a bomme ba ikutlwa ba sireletsegile ba sa dirise sekausu ka nako ya tlhakanelo dikobo?

**Phetogo mo maitsholong a tlhakanelo dikobo**

Borre mo motseng wa lona ba fetotse jang maitshwaro a bone a tlhakanelo dikobo ka lebaka la loaro la bonna?

GWETLHA: A go lebega ba tla fokotsa tiriso ya sekausu? A ba tla oketsa tiriso ya sekausu? Ka go reng?

A borre ba ba dirileng loaro la bonna ba dumela gore go siame go amogela tlhakanelo dikobo go sa dirisiwe sekausu ka gore ba dirile loaro la bonna?

A bomme ba ba lekanang le wena ka dingwaga ba gololesegile go kopa bakapelo ba bone ba ba dirileng loaro la bonna go dirisa sekausu? Fa mokapelo a sa dira loaro la bonna gone?

Botsalano (jwa nakwana, go ratana, kgotsa manyalo) a fetogile jang ka lebaka la loaro la bonna?

GWETLHA:

A go lebega borre ba ka oketsa go nna le bakapelo ba ba farologanyeng? A go lebega ba ka fokotsa?

A go lebega borre ba ka oketsa go nna le bakapelo ba ba farologanyeng ka nako e le nngwe? A go lebega ba ka fokotsa?

A bakapelo ba gololesegile kgotsa ga ba a gololesega go buisana ka seemo sa mogare wa HIV le ditsela tse di babalesegileng tsa tlhakanelo dikobo?

**Tse di amang go dira loaro la bonna ke borre**

- **Mabaka a go dira loaro la bonna (tse di rotloetsang)**

Ke eng borre ba ba lekanang le wena ka dingwaga ba itlhophela go dira loaro la bonna?

GWETLHA:

Go itshireletsa kgatlhanong le go fetelwa ke mogare wa HIV?

Mokapelo o batla gore rre a dire loaro la bonna?

Thotoetso go tswa mo go ba losika le ditsala?

Morafe o eme nokeng go dira loaro la bonna?

Bophepa?

Go kgatlhegela tebego ya bonna fa go dirilwe loaro la bonna?

- **Mabaka a go sa dira loaro la bonna (tse di kgoreletsang)**

Ke eng borre ba ba lekanang le wena ka dingwaga ba itlhophela go sa dira loaro la bonna?

GWETLHA:

Ga ba itse gore ditirelo tsa go dira loaro la bonna di teng kgotsa maduo a go dira loaro la bonna?

Ga ba kgone go bona nako go tswa ko tirong?

Ga ba kgone madi a dipalamo go ya le go bowa ko kokelong?

Ba tshaba botlhoko ka nako ya loaro le morago ga lone?

Ba tshoga gore loaro le ka tsala go fokotsega ga monate fa go tlhakanelwa dikobo?

Lebaka la go fola?

Bomme ba na seabe se le kae mo go rotloetseng borre go dira loaro la bonna?

GWETLHA: Bomma ba ka thusa jang go oketsa dipalo tsa borre ba ba dirang loaro la bonna?

Fa rre a batla go dira loaro la bonna, o ya itse jang gore o tshwanetse go ya kae?

GWETLHA: Mpolelela ka thuto-phatlatsa ya loaro la bonna mo motseng wa lona. Ke eng se se berekang sentle (kgotsa se se sa berekeng gotlhelele) e le bontlha bongwe jwa jwa thuto-phatlalatsa e e direlwang go oketsa dipalo tsa borre ba bagolo go diriwa loaro la bonna.

GWETLHA gape: ipapatso ya Television?

Ipapatso ya Radio?

Ketelo ko malapeng ka leloko la motse?

**Bokhutlo:**

A go na le sengwe gape se o eletsang go se buwa pele ga re fetsa puisanyo ya rona? Ke go lebogela go dumela go tsaya karolo mo puisanyong e. Go tshwaela ga gago go tla a thusa sechaba mo ntweng kgatlhanong le mogare wa HIV le bolwetse jwa AIDS, ebile go tla nna le seabe mo go tlhaloganyeng se loaro la bonna lo ka se dirang le ka fa re ka toka fatsang ka teng lenaneo la loaro la bonna lo lo babalesegileng mo Botswana.

**COMMUNITY LEADERS**

**Ithokotso (warm up)**

O utlwile eng ka loaro la banna lo lo diriwang mo bagolong?

GWETLHA: Kgolagano ya loaro la bonna le HIV?

Kgolagano ya loaro la bonna le malwetse a tlhakanelo dikobo?

Madup a loaro la bonna mo go bo mme?

**Maikutlo, Ditumelo, le Dikakanyo ka loaro la bonna**

Ke dingwaga dife tse di siametseng gore monna o ka dira loaro la bonna?

GWETLHA:

Ka go reng?

Dingwaga di ama jang gore a rre o dirile kgotsa ga a dira loaro la bonna?

A borre ba ba lekanang le wena ka dingwaga ba ka kgatlhegela go dira loaro la bonna fa ba ne ba ka rotloediwa go le dira ba le bannye?

Loaro la bonna le tsewa/lebiwa jang mo morafeng wa lona?

GWETLHA: Borre ba a reng fa ba buwa ka loaro la bonna le borre ba bangwe?

Bomme bone ba reng?

Batho ba mo motseng ba dira jang fa ba ka utlwa gore rre/monna o dirile loaro la bonna?

Matshwenyego a borre a matona ke eng pele ga ba dira loaro la bonna?

GWETLHA: Loaro/go segwa?

Ditlamorago tse di sa siamang tse di ka nnang morago ga loaro?

Matshwenyego a, amang jang go tsaya tshwetso ga rre go dira loaro loaro la bonna?

**Phetogo mo maitsholong a tlhakanelo dikobo**

Borre mo motseng wa lona ba fetotse jang maitshwaro a bone a tlhakanelo dikobo ka lebaka la loaro la bonna?

GWETLHA: A go lebega ba tla fokotsa tiriso ya sekausu? A ba tla oketsa tiriso ya sekausu? Ka go reng?

A borre ba ba dirileng loaro la bonna ba dumela gore go siame go amogela tlhakanelo dikobo go sa dirisiwe sekausu ka gore ba dirile loaro la bonna?

Ba raya bakapelo ba bone ba reng fa ba sa battle go dirisa sekausu?

A bomme ba ba lekanang le wena ka dingwaga ba gololesegile go kopa bakapelo ba bone ba ba dirileng loaro la bonna go dirisa sekausu? Fa mokapelo a sa dira loaro la bonna gone?

Botsalano (jwa nakwana, go ratana, kgotsa manyalo) a fetogile jang ka lebaka la loaro la bonna?

GWETLHA:

A go lebega borre ba ka oketsa go nna le bakapelo ba ba farologanyeng? A go lebega ba ka fokotsa?

A go lebega borre ba ka oketsa go nna le bakapelo ba ba farologanyeng ka nako e le nngwe? A go lebega ba ka fokotsa?

A bakapelo ba gololesegile kgotsa ga ba a gololesega go buisana ka seemo sa mogare wa HIV le ditsela tse di babalesegileng tsa tlhakanelo dikobo?

**Tse di amang go dira loaro la bonna ke borre**

- **Mabaka a go dira loaro la bonna (tse di rotloetsang)**

Ke eng borre ba ba lekanang le wena ka dingwaga ba itlhophela go dira loaro la bonna?

GWETLHA:

Go itshireletsa kgatlhanong le go fetelwa ke mogare wa HIV?

Mokapelo o batla gore rre a dire loaro la bonna?

Thotoetso go tswa mo go ba losika le ditsala?

Morafe o eme nokeng go dira loaro la bonna?

Bophepa?

Go kgatlhegela tebego ya bonna fa go dirilwe loaro la bonna?

- **Mabaka a go sa dira loaro la bonna (tse di kgoreletsang)**

Ke eng borre ba ba lekanang le wena ka dingwaga ba itlhophela go sa dira loaro la bonna?

GWETLHA:

Ga ba itse gore ditirelo tsa go dira loaro la bonna di teng kgotsa maduo a go dira loaro la bonna?

Ga ba kgone go bona nako go tswa ko tirong?

Ga ba kgone madi a dipalamo go ya le go bowa ko kokelong?

Ba tshaba botlhoko ka nako ya loaro le morago ga lone?

Ba tshoga gore loaro le ka tsala go fokotsega ga monate fa go tlhakanelwa dikobo?

Lebaka la go fola?

Bomme ba na seabe se le kae mo go rotloetseng borre go dira loaro la bonna?

GWETLHA: Bomma ba ka thusa jang go oketsa dipalo tsa borre ba ba dirang loaro la bonna?

Re itse gore go dira loaro la bonna mo go borre ba ba bagolo go ko tlase. O akanya gore ke eng borre ba ba bagolo (ba dingwaga tse di fetang masome a mararo) ba sa dire loaro la bonna?

GWETLHA:

Bagogi ba motse wa lona ba ka thusa jang go oketsa dipalo tsa borre ba ba dirang loaro la bonna?

Fa rre a batla go dira loaro la bonna, o ya itse jang gore o tshwanetse go ya kae?

GWETLHA: Mpolelela ka thuto-phatlatsa ya loaro la bonna mo motseng wa lona. Ke eng se se berekang sentle (kgotsa se se sa berekeng gotlhelele) e le bontlha bongwe jwa jwa thuto-phatlalatsa e e direlwang go oketsa dipalo tsa borre ba bagolo go diriwa loaro la bonna.

GWETLHA gape: ipapatso ya Television?

Ipapatso ya Radio?

Ketelo ko malapeng ka leloko la motse?

**Bokhutlo:**

A go na le sengwe gape se o eletsang go se buwa pele ga re fetsa puisanyo ya rona? Ke go lebogela go dumela go tsaya karolo mo puisanyong e. Go tshwaela ga gago go tla a thusa sechaba mo ntweng kgatlhanong le mogare wa HIV le bolwetse jwa AIDS, ebile go tla nna le seabe mo go tlhaloganyeng se loaro la bonna lo ka se dirang le ka fa re ka toka fatsang ka teng lenaneo la loaro la bonna lo lo babalesegileng mo Botswana.
